# Future Flood Risk Assessment under the Effects of Land Use and Climate Change in the Tiaoxi Basin

**DOI:** 10.3390/s20216079

**Published:** 2020-10-26

**Authors:** Leilei Li, Jintao Yang, Jin Wu

**Affiliations:** 1 State Key Laboratory of Desert and Oasis Ecology, Xinjiang Institute of Ecology and Geography, Chinese Academy of Sciences, Urumqi 830011, China; 2 University of Chinese Academy of Sciences, Beijing 100039, China; yangjt@lreis.ac.cn (J.Y.); wuj.17b@igsnrr.ac.cn (J.W.); 3 State Key Laboratory of Resources and Environment Information System, Institute of Geographic Sciences and Natural Resources Research, Beijing 100101, China

**Keywords:** NEX-GDDP dataset, CA-Markov model, SWAT model, Taihu basin, flood risk

## Abstract

Global warming and land-use change affects runoff in the regional basin. Affected by different factors, such as abundant rainfall and increased impervious surface, the Taihu basin becomes more vulnerable to floods. As a result, a future flood risk analysis is of great significance. This paper simulated the land-use expansion and analyzed the surface change from 2020 to 2050 using the neural network Cellular Automata Markov (CA-Markov) model. Moreover, the NASA Earth Exchange Global Daily Downscaled Climate Projections (NEX-GDDP) dataset was corrected for deviation and used to analyze the climate trend. Second, the verified SWAT model was applied to simulate future runoff and to analyze the future flood risk. The results show that (1) land use is dominated by cultivated land and forests. In the future, the area of cultivated land will decrease and construction land will expand to 1.5 times its present size. (2) The average annual precipitation and temperature will increase by 1.2% and 1.5 degrees from 2020 to 2050, respectively. During the verified period, the NSE and r-square values of the SWAT model are greater than 0.7. (3) Compared with the historical extreme runoff, the extreme runoff in the return period will increase 10%~25% under the eight climate models in 2050. In general, the flood risk will increase further under the climate scenarios.

## 1. Introduction

With the progress of global warming, temperature has risen in most basins of China and precipitation has become more uneven in the spatial-temporal distribution. The rapid changes in climate have caused increasingly prominent water problems, such as floods and drought, which can bring about a series of ecological, environmental, and social security problems [1]. Human interference, which influences river streams and floodplains, is also a cause of flash flood generation [2]. Because of the upper mountains, lower plains, and intensified human activities, the Tiaoxi basin has become a flood-prone area. There were six flood events from 1999 to 2018, two-thirds of which were caused by strong typhoons, and one-third caused by continuous heavy rainfall in the Tiaoxi basin. Super-typhoon Lichma passed through this basin with a speed of 20 km/h on 10 August 2019, making the water level rise and exceed the warning level rapidly. Many floods were caused by heavy rainfall during the rainy season or typhoon season, resulting in great economic losses. 

Previous studies have shown that land-use and climate change are the main factors that affect runoff and cause floods [3,4,5,6,7]. Urbanization can lead to an increase in impervious areas and shorten the concentrated time of direct runoff in the drainage network, resulting in a rapid increase in runoff [8,9]. Hollis has shown that the urbanization of a catchment can drastically change the flood characteristics of river and increase small flood flows [10]. Areu-Rangel has confirmed that the change of land use in the Villahermosa catchments is the main factor that explains the increase in inundation events observed over recent years [11]. In addition, natural water bodies (such as lakes, wetlands, and rivers) that can hold large amounts of water have been greatly reduced or filled, increasing the possibility of flooding [12]. Goonetilleke shows that urbanization significantly impacts water environments with increased runoff and the degradation of water quality [13]. Furthermore, McColl and Wang integrated a land-use forecasting model with the calibrated hydrologic model to improve land-use policy formulation at the watershed scale [14]. Meanwhile, climate change can affect rainfall and change flood peaks. Faccini found that the risk of flash floods increased because of an increase in rainfall regime and associated increase in discharge [15]. Based on twelve GCM outputs under historical, Mou has shown that the frequency of simultaneous floods as well as that of extreme heavy precipitation events would augment in the Huaihe River of China [16]. Therefore, future land-use and climate simulation can be used as input data for the hydrologic model to assess flood risk.

Land-use change simulation, which originates from urban expansion, is mainly calculated by the cellular automata (CA) model. Based on the decision tree CA-Markov model, Li Xia makes full use of spatial variables to simulate land-use conversion rules and successfully analyze the dynamic expansion of urban land in the Pearl River Basin [17]. Pandey uses CA and the Markov model to simulate a large river basin and to help protect natural vegetation and the management of the built-up area [18]. An artificial neural network (ANN), which is integrated with the CA-Markov model to optimize and allocate urban expansion cells, shows more satisfactory results in urban areas [19]. Because the neural network algorithm has non-linear characteristics in simulating the conversion rules of different land-use types, it has a wide range of applications to simulate land use [20].

Climate model datasets, which are derived from general circulation model (GCM), can provide future rainfall and temperature. The evaluation of the Coupled Model Intercomparison Project Phase 5 (CMIP5) climate dataset shows that temperature and rainfall can be well simulated in the Yangtze basin and Qinghai-Tibet Plateau [21,22,23]. Since the dataset is similar to the actual situation, CMIP5 is suitable for large regional climate change simulation. Booij have shown that the average and extreme discharge behavior at the basin outlet become somewhat better with increasing climate model resolution by three hydrological model in the calibration and validation [24]. The NEX-GDDP dataset is comprised of downscaled climate scenarios, which are derived from CMIP5 and published by NASA in 2015. Comparing the dataset with CMIP5, Chen recently has shown that the accuracy of NEX-GDDP’s downscaling rainfall and temperature is significantly higher and more reliable than CMIP5 in China [25]. More coastal basins in China have frequently gone through extreme heavy rainfall events in recent years. Therefore, it is reasonable to use NEX-GDDP dataset and to assess the impact of climate change on flood risk in the basin.

With the development of global hydrology, future risk assessments have made great progress [26,27,28]. Due to large damages incurred under flood periods, conducting the flood risk assessment under climate change is important to reducing the damage of lives and property [29,30,31]. The soil water assessment tool (SWAT) model, widely used for runoff assessment, has been applied to examine the impact of different climate scenarios on the hydrologic processes in the Mississippi River basin [32]. The variable infiltration capacity (VIC) model has been used to generate runoff under various climate, hydrologic, and water resource scenarios. The HEC-HMS model, which was developed by the US Army Corps of Engineers, has been applied to assess runoff, simulate hydrologic events, and forecast short-term flooding in different basins [33,34,35]. The SWAT model is used to explore the influences of climate change on hydrology processes, simulate long-term runoff changes, and return a favorable performance in the future [36]. The hydro-climatic modeling allows us to predict the influences of climate change on floods and provide early warnings [37,38].

This article analyzes the effects of climate changes and land-use expansion on the hydrological processes in the Tiaoxi basin. Specifically, the SWAT model was used to simulate runoff and estimate the peak flow under different climatic and land-use scenarios [39,40,41,42]. The RCP 45 and RCP85 models in the NEX-GDDP climate datasets represent moderate and extreme scenarios of greenhouse gas emissions. This article takes the Tiaoxi basin of China as the study area, discusses the changes of extreme precipitation through the NEX-GDDP dataset, comprehensively calculates underlying land- use and flow changes, and finally assesses flood risks from 2020 to 2050. This paper is structured as follows. The study site and methodology are described in Section 2. The results are described in Section 3. A discussion is offered in Section 4 and the conclusion is provided in Section 5.

## 2. Materials and Methods 

### 2.1. Study Area

**Figure 1:**
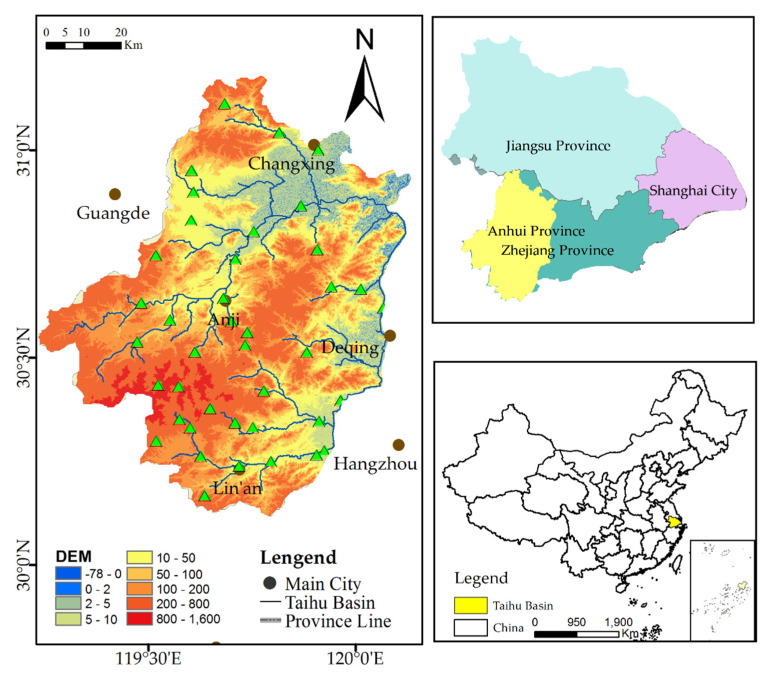
The geographic location of the Tiaoxi basin.

As shown in Figure 1, the Tiaoxi basin is an upstream sub-watershed of the Taihu basin in the northern section of Zhejiang Province, which also belongs to the Yangtze basin in China. The population is 1.23 million and the GDP is 25 billion RMB, which means that if there is a flood, there will be great losses. With a main channel of 158 km and an area of 4576 km^2^, the Tiaoxi basin is composed of the eastern and western Tiaoxi basin, which together flow into the central lake.

From the perspective of terrain, the Tiaoxi basin originates from Tianmu Mountain, whose downstream area is the Hangjiahu Plain, with a drop of 779 m. The Tiaoxi basin is bordered by the Dangtang Embankment to the east and the mountain ridgeline to the west. The Tiaoxi basin has dense artificial rivers, numerous reservoirs, and large lakes, the largest of which covers an area of 2338 km^2^. The overall pattern of the river network is dense, crisscrossed, and surprisingly similar to urban road networks in the Hangjiahu plain. About 80% of the area includes shrubs and fertile cultivated land in this basin. The Tiaoxi basin is dense in population and rich in wealth.

With an overall average rainfall total of 1177 mm, average annual rainfall gradually decreases from 1408 mm in the southwest Tianmu Mountains to less than 1100 mm in the eastern coastal region and the northern plain. From May to July, the basin is in the continuous rainfall period, with a range of 450–510 mm. From August to September, it is in the short-lived typhoon period, with a range of 190–380 mm. This rainfall accounts for about 75% of the annual total from May to September. Due to a series of factors, such as abundant rainfall, dense river networks, flat terrain, increased impervious surfaces, and tides, the flood problem is prominent and seriously endangers people’s lives and property. Therefore, it is of great significance to make precise flood-risk assessments for future local conservation.

### 2.2. Data Source

#### 2.2.1. DEM Data

With approximately 12 m spatial resolution and GCS_WGS_84 geographic coordinates, the ALOS DEM data were produced from the Japan PRISM sensor in 2006 and extracted to the Tiaoxi basin. The ALOS DEM can be downloaded from the website [43]. The entire DEM is composed of 23 small DEM images in the Tiaoxi basin.

#### 2.2.2. Soil Data

The 1:100,0000 scale soil classification data were collected from the Resource and Environment Data Center of the Chinese Academy of Sciences in 2008 and extracted to the study basin. The soil is occupied by yellow soil, red soil, yellow-red soil, brown-red soil, paddy soil, coarse soil, and limestone soil in the study area. In total, paddy soil has the largest distribution area, accounting for 44.8% of the total. Yellow-red soil follows, with a proportion of 9.8%.

#### 2.2.3. Land Use

The datasets showing land use in 2005, 2010, and 2017 were downloaded and extracted to the study area from Tsinghua University website [44]. With an overall accuracy of 72.76%, the datasets were produced by Gong Peng’s research group from the global 30m-resolution land use and 10 m-resolution Sentinel-2A images. The land cover is divided into nine categories: farmland, woodland, grassland, shrubs, wetlands, water bodies, impervious surfaces, bare land, and snow. Farmland and woodland are the main types, accounting for 40.6% and 32.6% in the study area, respectively.

#### 2.2.4. Gauge Data

The flow data, which were obtained from 54 monitoring stations of the local Taihu hydrological bureau, mainly include daily flow data from 1999 to 2019. The measured rainfall data, which were downloaded from the China Meteorological Data website, come from the daily V3 dataset interpolated from China’s surface meteorological data with a time range of 1999 to 2019.

#### 2.2.5. NEX-GDDP Data

NEX-GDDP is a high-resolution downscaling daily dataset, composed of 21 coupling modes of CMIP5 and published by NASA [45,46]. NEX-GDDP is downloaded and extracted from the Google Earth Engine platform for the extent of the basin. The NEX-GDDP downscaling dataset includes the RCP4.5 and RCP8.5 emission scenarios with a time range of 1966 to 2099. RCP45 is a stable pathway that peaks CO_2_ at 4.5 W/m^2^, while RCP85 is the worst scenario with rising radiative forcing of 8.5 W/m^2^ [47,48]. Each climate model dataset includes historical precipitation and temperature from 1950 to 2005 and predictive precipitation and temperature from 2006 to 2099.

With the 0.25-degree spatial resolution in the NEX-GDDP dataset, a total of 20 grid points are included inside the Tiaoxi basin. The improved spatial resolution and data reliability better predict the future climate than does the CMIP5 dataset [49]. Based on historical research, the precipitation datasets in the eight models of NEX-GDDP are verified and indicate that extremely high consistency with the actual situation in China [50]. The NEX-GDDP dataset can help analyze regional climate change and deal with future evolution under extreme climate conditions in the basin. The detailed climate model is shown in Table 1.

### 2.3. Processing Methods

**Figure 2:**
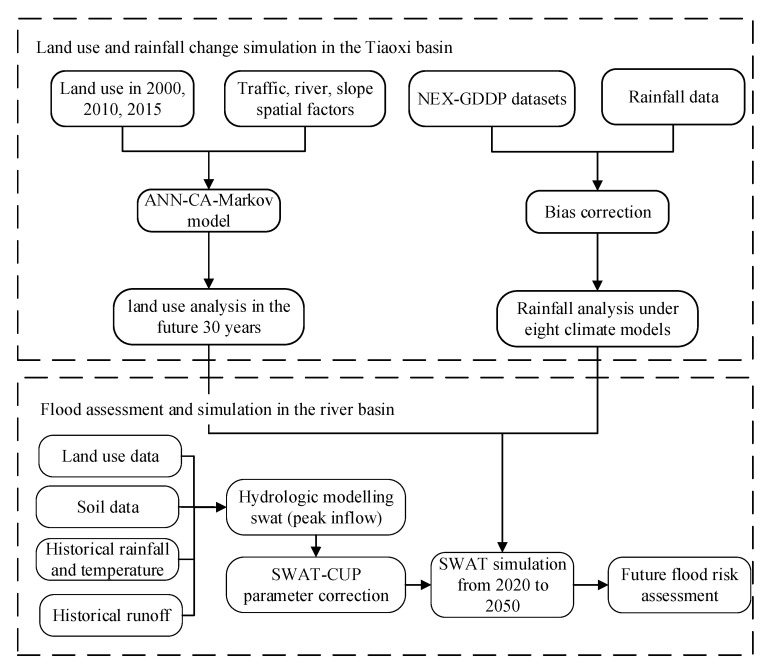
The overall method of flood risk assessment in this study.

The overall approach used for the future flood risk assessment of the Tiaoxi basin is shown in Figure 2. It mainly includes four steps. First, the CA-Markov model and many spatial factors are used to estimate land-use change from 2020 to 2050. Secondly, historical rainfall and NEX-GDDP datasets are used for bias correction by the CMhyd tool. Thirdly, the SWAT model is analyzed and verified using historical measured rainfall, temperature, and flow data. Finally, eight kinds of climate models in NEX-GDDP and the verified SWAT model are used to predict monthly runoff and to assess future flood risk from 2020 to 2050 in the Tiaoxi basin.

#### 2.3.1. CA-Markov Model Predicts Land Use Change

CA-Markov model combines cellular automata and Markov chain to predict the land use trends and characteristics over time [51]. CA, which has powerful spatial modeling and computing capabilities, usually divides the simulated geographic space into uniform regular grids, and successively calculates the state changes between different grids over time, and finally forms a simulation of land use changes. The status of CA-Markov model’s regular grids is usually determined by the attribute types of land use, such as farmland, woodland, grassland, water bodies, and construction land, etc. When there are large numbers of grids with same certain land-use type in the four or eight neighborhoods, the middle grid has a higher probability of evolving into this type [52,53]. The transition of each grid in CA-Markov model is greatly affected by spatial distance variables. The expansion of land use is closely related to a series of spatial variables such as distance from the city center, distance from the transportation network, slope, DEM elevation and other environmental factors. Previous research has shown the transition of each grid state is determined by a function composed of these variables, including system dynamics (SD), neural network, decision tree, and logistic regression methods. Because of the strong nonlinear simulation ability and high simulation accuracy in ANN-CA-Markov model than other methods, we chose this model to simulate the expansion of land use from 2020 to 2050 [54].

The detailed roadmap of land use expansion is shown in Figure 2. First, land use and eight spatial variables are resampled to the same row and column numbers during data processing. Based on land use in 2000 and 2010, the MLP neural network module in the IDRISI 17 software is used to train land use transfer probability. The CA-Markov model calculates the transfer matrix from land use in 2000 and 2010, simulates the land use result in 2015, compares the results with real land use in 2015, and verifies the kappa accuracy of the ANN-CA-Markov model. Cohen’s kappa coefficient was generated from a statistical test to evaluate the accuracy of a classification in Equation (1). Po represented the ratio of the correct classification of each category to the total sample.

(1) $$Kappa=\frac{P_{o}-\frac{1}{N^{2}}\sum_{i=1}^{M}n_{i+}*n_{+i}}{1-\frac{1}{N^{2}}\sum_{i=1}^{M}n_{i+}*n_{+i}}$$

**Figure 3:**
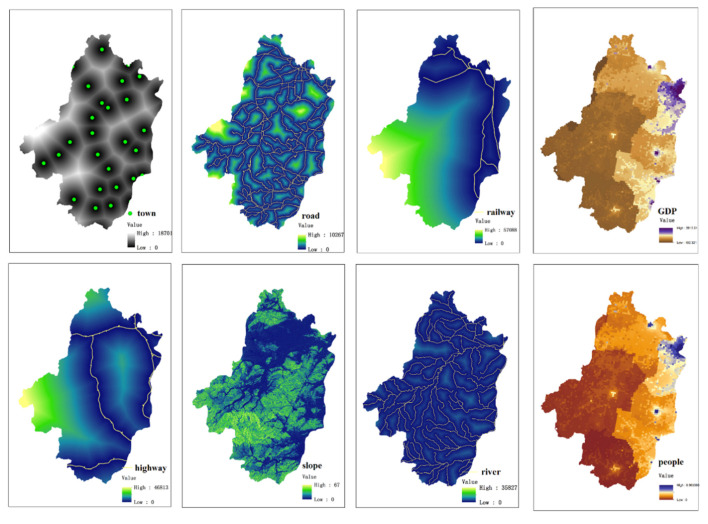
The spatial variables for the CA-Markov simulation in land use.

Further 30-year land use from 2020 to 2050 can be predicted by the ANN-CA-Markov model. As shown in Figure 3, the normalized spatial variables for land use simulation include: (1) distance from the river; (2) distance from the town center; (3) distance from the road; (4) distance from the highway; (5) distance from the railway; (6) slope data; (7) population density and (8) GDP density.

#### 2.3.2. NEX-GDDP Rainfall Data Correction

Generally, the NEX-GDDP rainfall data have a large deviation in frequency and intensity [55]. Under the models BCC_CSM_1, BNU_ESM, CCSM4, CSIRO_MK3_6_0, GFDL_ESM2G, IPSL_CM5A_MR, and MRI_CGCM3 in the NEX-GDDP dataset, this study used historical observations of rainfalls to verify the deviation and correct the rainfall values in eight climate models. Historical observations of rainfall were borrowed from the daily surface V3 dataset of China’s surface meteorological data from 2000 to 2006. The CMhyd tool includes seven available bias-correction methods, which are distribution mapping, linear scaling, delta-change correction, local intensity scaling, and power transformation, etc. Based on the overlapping years between historical precipitation and climate model precipitation from 2000 to 2006, a precipitation local intensity scaling method reveals the best-satisfied correlation and is chosen to correct future precipitation from 2020 to 2050. The bias correction method is used to calculate the maximum and minimum on the distribution frequency so that the predicted rainfall is relatively consistent with actual values in the eight climate models.

**Figure 4:**
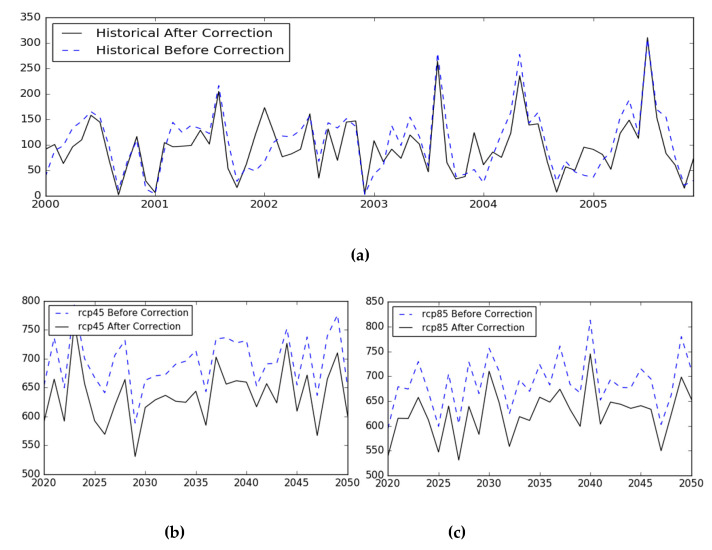
(**a**) The corrected comparison of eight models using the local intensity scaling method. (**b**) The bias correction of future average rainfall in eight models under the RCP45 scenario. (**c**) The bias correction of future average rainfall in eight models under the RCP85 scenario.

The correction process of the rainfall data contains two parts: the verification in the historical period and the correction in the future period. Figure 4a shows the parameter verification between historical rainfall in eight climate models and historical observations of rainfall using the local intensity scaling method of CMhyd. Due to the discrepancy between the historical and future rainfall in the eight climate models, this paper corrects the rainfall under the RCP45 and RCP85 scenario by the CMhyd tool, as shown in Figure 4b,c. The bias corrected effects of future rainfall in eight climate models are shown in Figure 4b,c. 

#### 2.3.3. Hydrological Simulation

The SWAT model is a continuous hydrological model used to verify monthly or annual historical runoff, simulate future runoff and predict the impact of the underlying surface and rainfall to runoff. The SWAT model divides the watershed into multiple sub-basins based on DEM, splits the sub-basins into several hydrological response units (HRU) according to different land cover and soil types, calculates the runoff of each HRU and aggregates it into a total runoff. The hydrological process of the SWAT model mainly includes two parts: runoff generation and slope confluence, and river confluence. The SWAT model is mainly affected by factors such as climate, soil, vegetation, and underlying surface. Meteorological variables include rainfall, temperature, solar radiation, wind speed, and relative humidity, which can be input by models or measured data. The SWAT model follows the water balance Equation (2).

(2) $$SW_{t}=SW_{0}+\sum_{i=1}^{t}\left(R_{day}-W_{surf}-E_{\alpha }-W_{seep}-Q_{gw}\right)$$

where *SW_t_* is the final soil moisture content; *SW*_0_ is the early soil moisture content; *t* is the time step; *R_day_* is the rainfall on the *i* day; *Q_surf_* is the surface runoff on the *i* day; *E_α_* is the evaporation on the *i* day; *W_seep_* is the amount of water entering the unsaturated zone from the soil profile on day *i*; *Q_gw_* is the ground water content on day *i*.

In this paper, the scheme of the SWAT model is mainly as follows: the SCS curve method is used for runoff calculation; the soil flow calculation selects the dynamic water storage method; and the river flow calculation selects the Muskingum method. The surface runoff of the HRU can be calculated by the SCS curve method, which assumes that the actual water storage capacity is equal to the ratio of the difference between the runoff and rainfall after soil is saturated to the maximum water storage. As shown in Equation (1), the SCS curve method is calculated by the CN value, which is closely related to the soil type, vegetation coverage, surface condition, vegetation type, and soil moisture in the study area. The soil flow is related to factors such as hydraulic conductivity, slope and soil moisture content. The dynamic water storage method is used to calculate the soil flow *Q_lat_* as shown in Equation (3). The ground water *Q_gw_* is calculated by the Equation (4).

(3) $$S=\frac{25400-254CN}{CN}$$

(4) $$Q_{lat}=0.024\times \left\{\frac{2SW_{ly,excess}\times K_{sat}\times slp}{\phi_{d}\times L_{hill}}\right\}$$

where *K_sat_* is the saturated hydraulic conductivity of soil, s*_lp_* is the slope, *S_Wly, excess_* is the amount of water that can flow out of soil saturated zone, *L_hill_* is the length of slope, and *d* is the total pore gap that can flow out of soil layer.

The hydrological SWAT and NEX-GDDP climate models can complete the entire process from rainfall to future flood risk assessment. First, the bias-corrected NEX-GDDP precipitation, DEM, land use, and soil data can be used as raw input data for hydrological SWAT models. Second, hydrological runoff curves are generated by the SWAT model from 2020 to 2050. SWAT-CUP is a software for calibrating SWAT models, which can be used to perform calibration, verification, sensitivity analysis and uncertainty analysis. Compared to historical observed flow, the SWAT-CUP is used to calibrate model parameters and improve the correction ability to make simulated runoff more reliable. The Nash-Sutcliffe efficiency (NSE) and coefficient of determination (R^2^), which are used to test the effect of simulation, can be calculate by Equations (5) and (6) [56]. Third, the corrected climate model rainfall and the SWAT model are used to simulate future flow in the Tiaoxi Basin. At last, the flood risk assessment is analyzed by the flood return period in the Tiaoxi Basin.

(5) $$NSE=1-\frac{\sum_{i=1}^{n}\frac{{\left(Q_{obsi}-Q_{modi}\right)}^{2}}{{\left(Q_{obsi}-\bar{Q_{obs}}\right)}^{2}}}{\sum_{i=1}^{n}{\left(Q_{obsi}-\bar{Q_{obs}}\right)}^{2}}$$

(6) $$R^{2}=\frac{1}{n}\sum_{i=1}^{n}{\left(Q_{obsi}-Q_{modi}\right)}^{2}$$

In formulas (5) and (6), *Q_obsi_* and *Q_modi_* indicate the observed runoff and simulated runoff, respectively. *Q_obs_* is the mean observed runoff. N is the number of observations. 

## 3. Results

### 3.1. Land-Use Change Analysis

**Figure 5:**
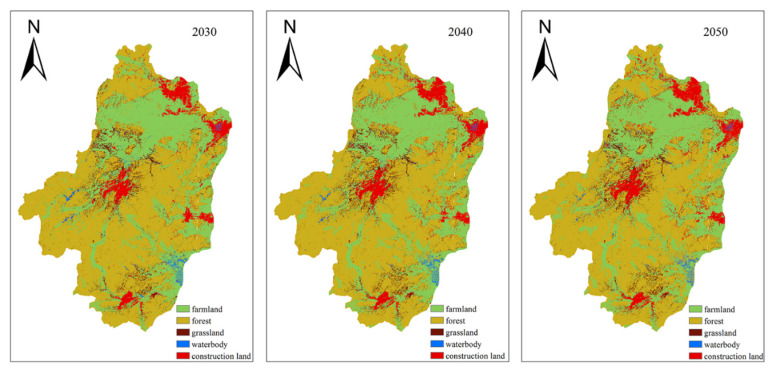
The simulated land use by CA-Markov model in 2030, 2040 and 2050.

As shown in Figure 5, this paper uses these spatial variables to calculate land conversion adaptability and the CA-Markov model to simulate land use in 2030, 2040, and 2050. During the verification period of the CA-Markov module, this paper takes the two-stage land use for the years 2000 and 2010 to simulate the 2015 land use through the IDRISI software. Compared to real land-use in 2015, it shows that the overall accuracy of Kappa reaches 85.3%, which means the simulated expansion is ideal.

**Figure 6:**
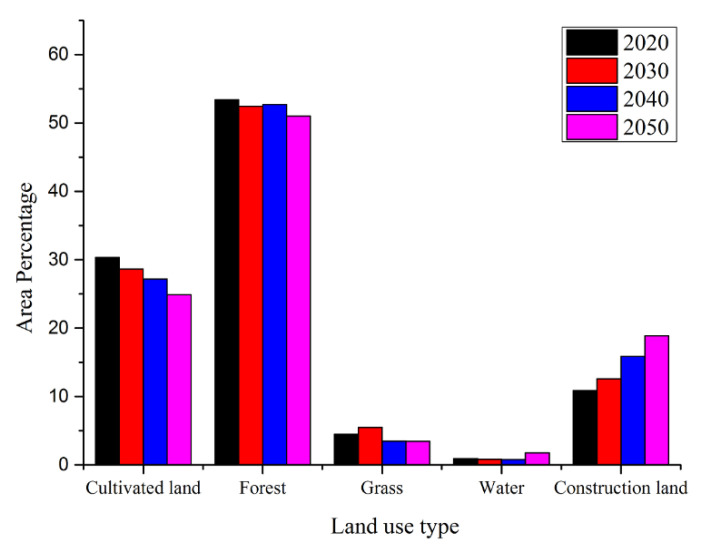
Land use change according to the CA-Markov model in 2030, 2040 and 2050.

According to the statistics, the land use is mainly composed of cultivated land, woodland, grassland, and construction land, accounting for 28%, 52%, and 15%, respectively, in 2050. Compared with land use in 2020, cultivated land will make a reduction of 280 km^2^ and a decrease of about 5.1% in 2050. As shown in Figure 6, the forest and waters are predicted to have few changes in 2050. The most obvious change is in construction land, with an increasing area of 392 km^2^, which is 1.5 times that of the area in 2020.

### 3.2. NEX-GDDP Rainfall Data Correction and Analysis

#### 3.2.1. Rainfall and Temperature Change Trend Analysis

**Figure 7:**
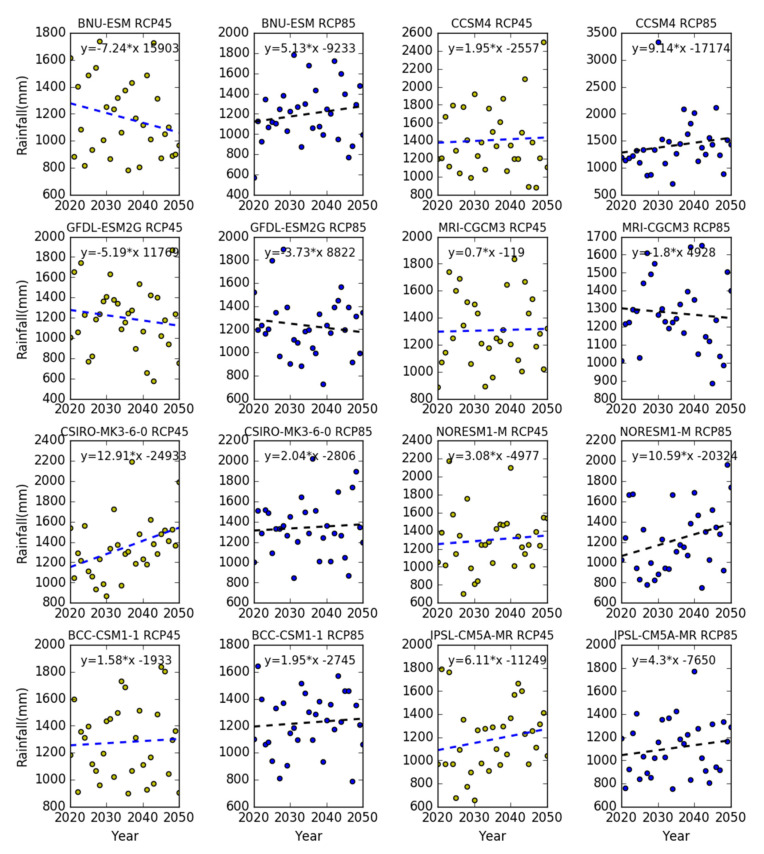
The time-change analysis of rainfall from 2020 to 2050 in the Tiaoxi basin.

Under the RCP45 and RCP85 scenarios, linear regression analysis of rainfall was performed in the BCC_CSM_1, BNU_ESM, CCSM4, CSIRO_MK3_6_0, GFDL_ESM2G, IPSL_CM5A_MR and MRI_CGCM3 models of NEX-GDDP dataset from 2020 to 2050. As shown in Figure 7, the average annual rainfall slowly increases, with a rate of 7 mm/10 years under the MRI-cGCM3 model. Average annual rainfall increases between 15.8 mm/10 years and 40 mm/10 years under the CCSM4, BBC-CSM-1, CSIRO-MK3-6-0, NORESM1-M and IPSL-CMSA-MR climate models. However, average annual rainfall shows a downward trend under the BNU-ESM and GFDL-ESM2G climate models. On the whole, the future rainfall is expected to increase, but not in obvious ways.

**Figure 8:**
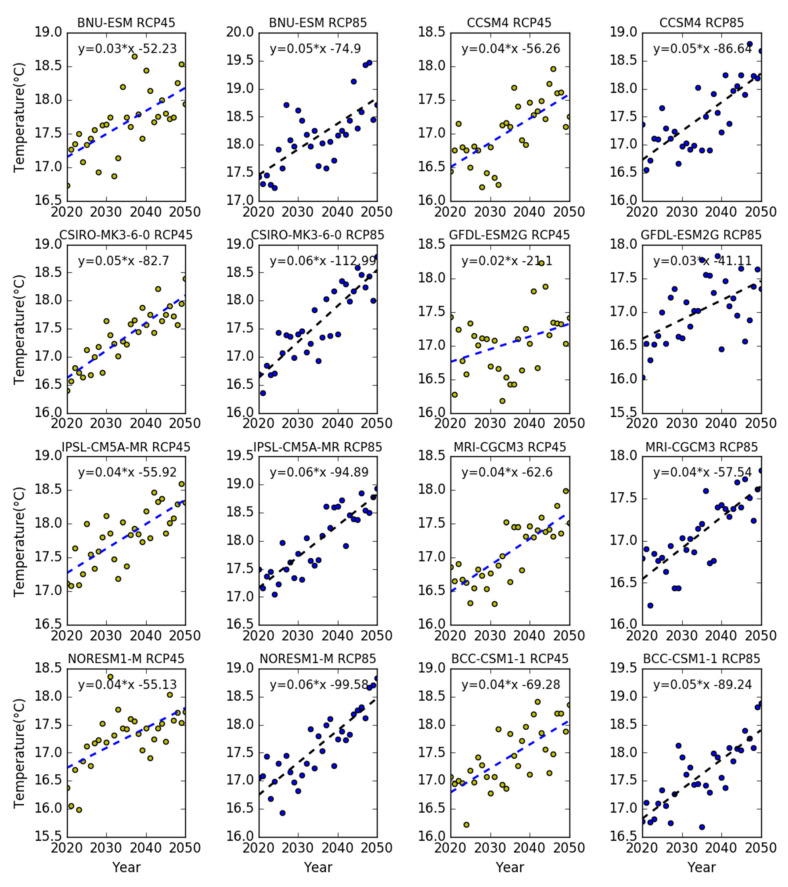
The temperature trend in the eight climate models from 2020 to 2050.

The future temperature will show a significant growth trend. Under the eight climate models in the RCP4.5 and RCP8.5 scenarios, annual average maximum and minimum temperatures will increase rapidly by 0.041 °C/year and 0.045 °C/year, according to the linear regression analysis conducted for the period 2010–2050. As shown in Figure 8, the average temperature will increase by 1.5 °C in the future 40 years. The temperature increase was more intense in the RCP85 scenario than in the RCP45 scenario.

#### 3.2.2. Analysis of Extreme Rainfall Trends

**Figure 9:**
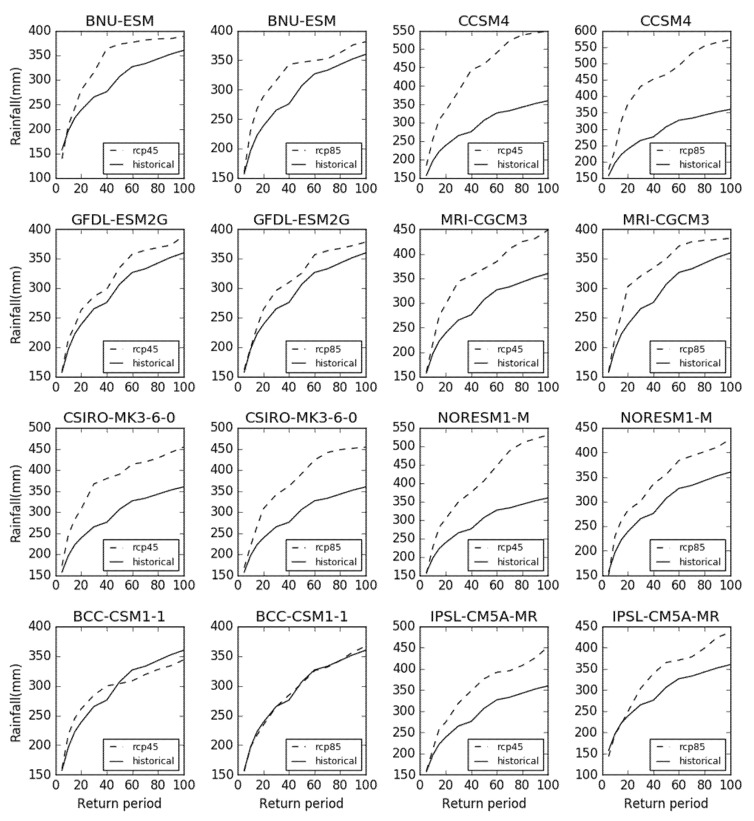
The extreme future rainfall of eight climate models in different return periods.

Although the average rainfall has not changed much, the extreme rainfall of the basin has a clear increasing trend. Under the BCC_CSM_1, BNU_ESM, CCSM4, CSIRO_MK3_6_0, GFDL_ESM2G, IPSL_CM5A_MR, MRI_CGCM3, and NorESM1_M climate models, this article predicts extreme rainfall frequency in the RCP45 and RCP85 scenarios from 2020 to 2050. Historical measured rainfall indicates that the extreme rainfall standard of once in 100 years is 350 mm. As shown in Figure 9, extreme rainfall in the BBC_CSM1_1 model has not changed significantly from 2020 to 2050 under the RCP45 and RCP85 scenarios. Under the RCP45 scenario, extreme rainfall frequency showed an increasing trend of 10–25% in the BNU_ESM, CCSM4, CSIRO_MK3_6_0, GFDL_ESM2G, IPSL_CM5A_MR, MRI_CGCM3, and NorESM1_M climate models. Under the RCP85 scenario, extreme rainfall also showed a steady increasing trend in the 10-, 20-, 50-, and 100-return periods. At the same time, extreme rainfall in the RCP45 scenario is slightly higher than that in the RCP85 scenario.

### 3.3. SWAT Model Verification and Simulation

Rainfall and runoff were verified by the SWAT model in the Tiaoxi basin. The parameters involve the basin structure, CN value, soil index, and basin lag in the SWAT rainfall-runoff model. As shown in Table S1, the SWAT model is calibrated by adjusting 13 model parameters, including R_CN2, V_ALPHA_BF, V_GW_DELAY, V_GWQMN, V_GW_REVAP, V_ESCO, V_CH_N2, V_CH_K2, V_ALPHA_BNK, R_SOL_AWC, R_SOL_K, R_SOL_BD, and V_SFTMP in the SWAT-CUP software. The number of simulation cycles is 300 to achieve a better simulation effect and to obtain the best parameter range in the SWAT-CUP software.

This regular verification process is performed by repeatedly adjusting the processing parameters manually to minimize the error between simulated and observed historical runoff. Figure S1 shows the comparison effect of the measured and simulated runoff. The Nash–Sutcliffe efficiency (NSE) and coefficient of determination (R^2^) are bigger than 0.7, which means that the calibrated SWAT model can accurately simulate historical runoff and produce satisfactory effects.

**Figure 10:**
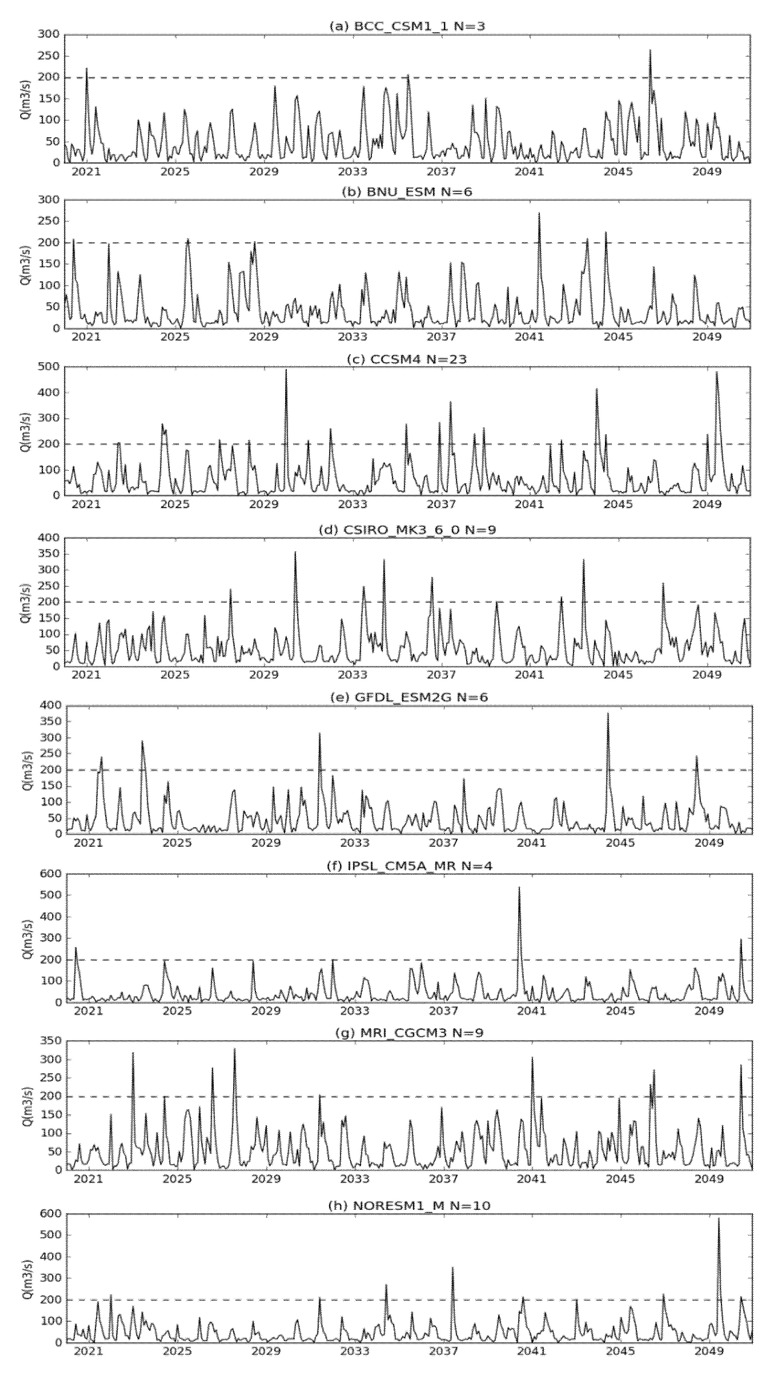
Future monthly runoff times exceeding the historical period under the eight climate models, including (**a**) BCC_CSM_1 (**b**) BNU_ESM (**c**) CCSM4 (**d**) CSIRO_MK3_6_0 (**e**) GFDL_ESM2G (**f**) IPSL_CM5A_MR (**g**) MRI_CGCM3 (**h**) NorESM1_M.

The long-term simulated runoff under eight climate models is shown in Figure 10. Including BCC_CSM1_1, BNU_ESM, CCSM4, CSIRO_MK3_6_0, GFDL_ESM2G, IPSL_CM5A_MR, MRI_CGCM3, and NorESM1_M climate models, the corrected rainfall is the main input variables of the SWAT model in the future runoff assessment. Based on the verified model parameters, the SWAT model is used to simulate future runoff from 2020 to 2050. The runoff of once in 100 years was 200 m^3^ in the historical period. As shown in Figure 10, the number of future simulated runoff instances exceeding 200 m^3^ is more than three times than the historical period. Meanwhile, extreme runoff is more serious in the CCSM4, CSIRO_MK3_6_0, MRI_CGCM3, and NorESM1_M climate models.

The extreme runoff analysis in different recurrence period is shown in Equation (7).

(7) T=1/(1−F(p,0))

where *T* represents the extreme runoff value in a certain period and *F* captures a point in the continuous distribution function, which corresponds to the probability *p*.

**Figure 11:**
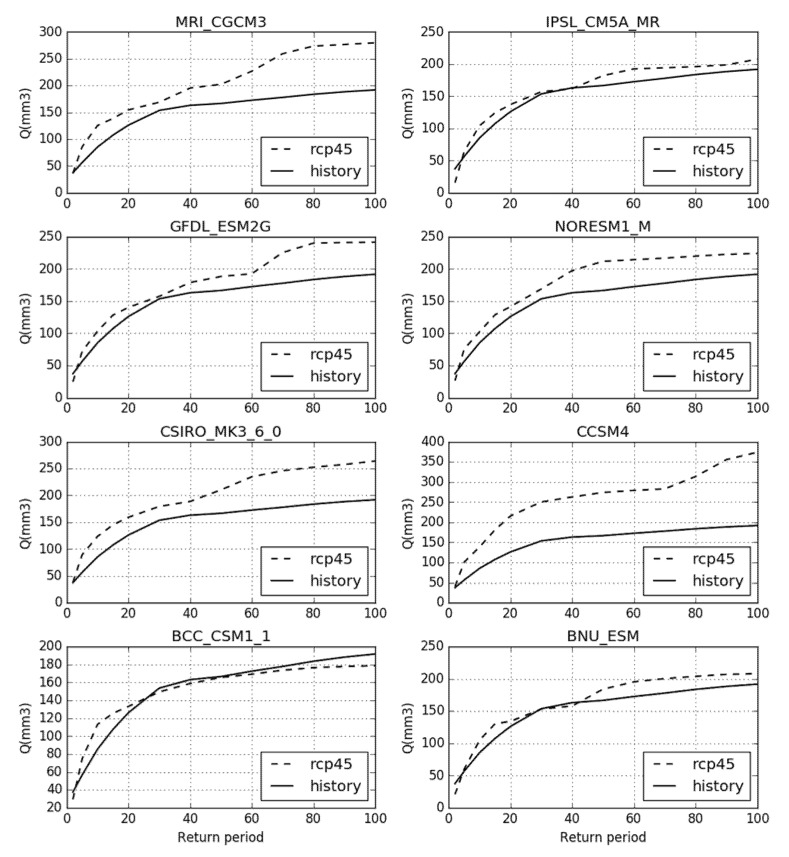
The extreme runoff standard in recurrence periods of 10, 20, 50, and 100 years.

The extreme runoff of different recurrence periods is calculated under the BCC_CSM_1, BNU_ESM, CCSM4, CSIRO_MK3_6_0, GFDL_ESM2G, IPSL_CM5A_MR, MRI_CGCM3, and NorESM1_M climate models in 2020–2050. As shown in Figure 11, runoff is stable in the BCC_CSM_1 climate model. Under the other seven models, the extreme runoff greatly increases between 10 years, 20 years, 50 years, and 100 years. Under the IPSL_CM5A_MR, NorESM1_M, and BNU_ESM climate models, the extreme runoff is predicted to by 10% during the recurrence period. Under the MRI_CGCM3, GFDL_ESM2G, CSIRO_MK3_6_0, and CCSM4 climate models, the extreme runoff increases from 200 m^3^/s to 250 m^3^/s during the recurrence period, an increase of over 25%. In general, the flood risk shows an increase from 2020 to 2050 in the Tiaoxi basin.

## 4. Discussion

### 4.1. Land Use Change

Based on the predicted occurrence probability, the CA-Markov model can simulate the conversion frequency among different land use types. However, the dynamic conversion requires multiple spatial variables to determine the CA-Markov model function. This paper combines the CA-Markov model with a neural network to simulate the complex land use evolution process. Artificial neural network and basin spatial variable factors are used to adjust the model parameters and structure, determine the conversion rules of land use types, and improve the accuracy of the CA-Markov model. According to the land use calculated by the CA-Markov model, it can be seen that the construction land shows a clear growth trend from 2030 to 2050. Because of the small proportion of construction land, this land use change will have little impact on the runoff from 2020 to 2050. The mountainous terrain in the watershed, which limits the land use expansion to a certain extent, leads to insignificant changes in simulated cultivated land and construction land. This conclusion is consistent with previous studies that suggest LUCC will not undergo huge structural changes [57,58].

### 4.2. Climate Change Trend in NEX-GDDP

The Tiaoxi basin is a small watershed located at the junction of land–sea and mountain–plain. The NEX-GDDP dataset, which covers 40 points in the study area, can be used to analyze the long-term trends in the small-scale basin from 2020 to 2050. Compared to the previous CMIP5 dataset, the NEX-GDDP dataset has an important advantage, with a spatial resolution of 0.25*0.25 degrees. Under the eight climate models in different scenarios, the annual average temperature also shows a significant increasing trend, with a rapid rise of 1.5 °C in the next 40 years. Although there are differences in rainfall under the BCC_CSM_1, BNU_ESM, CCSM4, CSIRO_MK3_6_0, GFDL_ESM2G, IPSL_CM5A_MR, MRI_CGCM3, and NorESM1_M climate models, extreme rainfall is constantly increasing under the once every 100, 50, 20 and 10 years. Based on the analysis of CMIP5, previous studies in the Yangtze River Basin also showed the temperature and rainfall are increasing in this area, which is consistent with the article’s conclusions from the NEX-GDDP dataset [59,60,61]. This shows that climate has a significant effect on runoff in the small-scale Tiaoxi basin.

### 4.3. Future Runoff Trend

This article uses eight climate datasets and the SWAT hydrological model to estimate the monthly runoff from 2020 to 2050 in the Tiaoxi basin. On this basis, the runoff frequency is used to analyze the extreme runoff in different recurrence periods under the RCP45 and RCP85 scenarios. Under the eight climate models, the results show that the estimated runoff in the period 2020–2050 is consistently greater than the baseline period of 2000–2019, with an average increase of 10~25%. Wu shows that high risks might be concentrated in the southern regions of the Yangtze River Basin from a perspective across China [62]. As a downstream sub-watershed of the Yangtze basin, we took the Tiaoxi watershed as the study area and proved the same point from the perspective of a local basin. This indicates that climate change will increase the flood risk. In the future, the design flows for different recurrence periods should be adjusted to accommodate climate change, which demands a higher capacity of structures, such as dams and river embankments, in the Tiaoxi basin. 

### 4.4. Uncertainty and Suggestions

With the continuous development of downscaling technology, high-resolution regional climate models are expected to improve the accuracy of regional further climate assessment and further flood risk assessment. We use the historical rainfall to correct eight climates datasets, which can reduce the errors and uncertainties introduced by the climate models to the local small-scale basin. Although the uncertainty of rainfall, temperature, and land use will lead to differences in runoff, the simulated flow in the corrected SWAT model shows that the flood risk is constantly increasing in multiple climate modes. With the improvement of human prediction toward climate systems in the future, the climate models analysis of NEX-GDDP will be more conducive to reducing uncertainty, predicting flood risk, and determining appropriate flood engineering design standards in long-term water resources planning.

## 5. Conclusions

This paper analyzes future flood risk under land-use expansion and climate scenario changes in the Tiaoxi basin. As land use and climate changes and their combined effects have become a pressing issue, our study quantified the future flood risk in these drivers under different scenarios in the Taihu Basin. The results are summarized as follows: (1) Deviation correction has a positive effect on reducing forecast rainfall errors. The average annual precipitation and temperature will increase by 1.2% and 1.5 degrees, respectively, from 2020 to 2050. (2) In the future, construction land will increase by 392 km^2^ in 2050, which is 1.5 times that of the land in use in 2020. Cultivated land will greatly reduce by 280 km^2^, which accounts for 5.1% of the total. (3) Under the BNU_ESM, CCSM4, CSIRO_MK3_6_0, GFDL_ESM2G, IPSL_CM5A_MR, MRI_CGCM3, and NorESM1_M climate models, the extreme runoff standard will increase by 10~25% over the historical period. In the next 30 years, the peak discharge and flood risk have a high probability of increasing in the Tiaoxi basin.

This conclusion indicated the effects of future land use and climate changes on flood risk, which is conducive to regional flood control planning and sustainable development under future predictions of climate change. Therefore, we recommend that policy makers in the Taihu Basin improve the water regulation and storage capacity of upstream river and lake network. Human activities and climate change exacerbate flood risk and affect the development of the basin in the future. Consequently, an important area for future research is reducing the uncertainty of the models and optimizing the structure of land use to ensure the sustainable development of a watershed.

## Figures and Tables

**Table 1:** Main climate model description in NEX-GDDP dataset.

Climate Model	Model Description	Resolution	Time Range
BCC-CSM1-1	Beijing Climate Center Climate System Model	0.25 × 0.25	2006–2099
BNU-ESM	Beijing Normal University Earth System Model	0.25 × 0.25	2006–2099
CCSM4	The Community Climate System Model	0.25 × 0.25	2006–2099
CSIRO-Mk3-6-0	the CSIRO-Mk3.6.0 Atmosphere Ocean Global Climate Model	0.25 × 0.25	2006–2099
GFDL-ESM2G	GFDL’s ESM2 Global Coupled Climate Model	0.25 × 0.25	2006–2099
IPSLCM5A-MR	Institute Pierre Simon Laplace Model CM5A-MR Climate Model	0.25 × 0.25	2006–2099
MRI-CGCM3	Meteorological Research Institute CGCM Climate Model	0.25 × 0.25	2006–2099
NorESM1-M	Norwegian Climate Centre Earth System Model	0.25 × 0.25	2006–2099

## Supplementary Materials

The following are available online at https://www.mdpi.com/1424-8220/20/21/6079/s1, Figure S1: Calibration effects between existing historical runoff and simulated runoff, Table S1: SWAT model parameter values.
