# Supplementary material for: Future Flood Risk Assessment under the Effects of Land Use and Climate Change in the Tiaoxi Basin
Source: Sensors (Basel). 2020 Oct 26;20(21):6079. doi: 10.3390/s20216079 (PMC7663716; doi:10.3390/s20216079)
Supplement: Supplementary file 1 [file sensors-20-06079-s001.pdf]

Table 1. SWAT model parameter values

Model Parameter	Parameter Description	Initial Range	Best Value
R_CN2	runoff curve number	-0.2-0.2	-0.124
V_ALPHA_BF	base flow coefficient	0-1	0.245
V_GW_DELAY	groundwater delay coefficient	30-450	352.7
V_GWQMN	groundwater runoff coefficient	0-2	0.55
V_GW_REVAP	groundwater re-evaporation	0-0.2	0.026
V_ESCO	soil transpiration compensation	0.8-1	0.915
V_CH_N2	main channel Manning coefficient	0-0.3	0.155
V_CH_K2	channel hydraulic conductivity	5-130	18.13
V_ALPHA_BNK	riverbank regulation base flow	0-1	0.39
R_SOL_AWC	available soil water	-0.2-0.4	-0.05
R_SOL_K	saturated hydraulic conductivity	-0.8-0.8	0.573
R_SOL_BD	soil density	-0.5-0.6	0.412
V_SFTMP	snowfall temperature	-5-5	-1.35

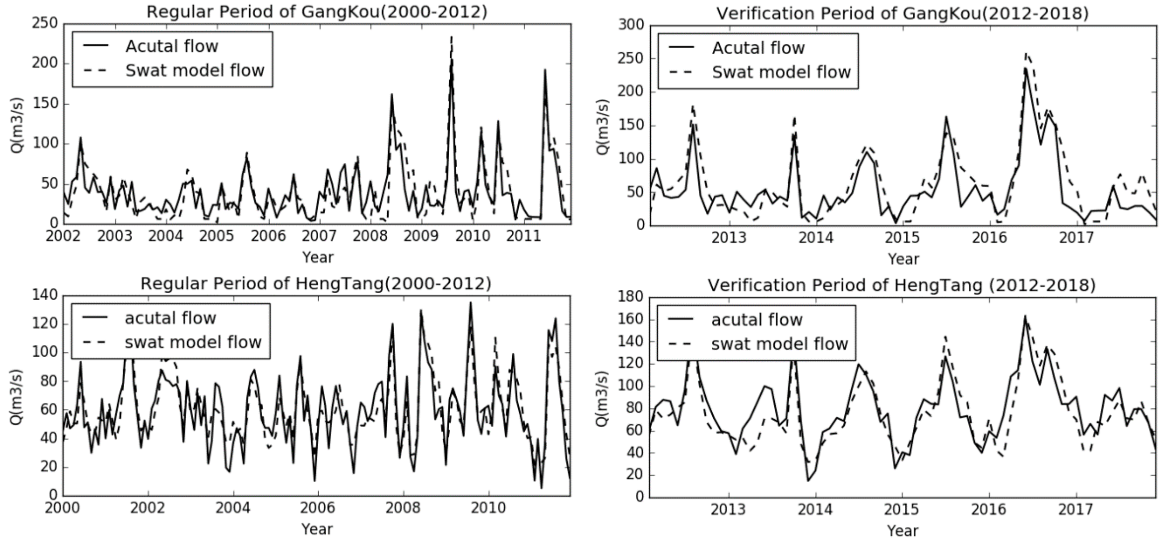

Figure 1. Calibration effects between existing historical runoff and simulated runoff
